# Social Determinants of Health in Urban Transgender Patients: A Case Report

**DOI:** 10.7759/cureus.42941

**Published:** 2023-08-04

**Authors:** Isaac Kim, Marta B Sekh, Tasmia Khan, Ravindi Gunasekara, Irina Kogan

**Affiliations:** 1 Psychiatry and Behavioral Sciences, Interfaith Medical Center, Brooklyn, USA; 2 Neurology, Interfaith Medical Center, Brooklyn, USA

**Keywords:** psychiatry case report, mental health and suicide, sdoh, mental health illness, psychiatry & mental health, mental health, lgbt+q, transgender health, trans health care, social determinants of health (sdoh)

## Abstract

The social determinants of health (SDOH) are a complex web of factors that influence the health of individuals throughout their lifetime. There are many drivers of health inequities within the SDOH, such as socioeconomic status, education, employment, gender, and race/ethnicity. It is possible that mental illness may develop when these factors negatively affect health. However, current research primarily focuses on SDOH in cisgender individuals leaving a scientific gap in transgender individuals who require unique considerations when providing comprehensive medical care.

We present the case of a 20-year-old transgender female who was admitted for suicidal attempts during a methamphetamine overdose, and who had been struggling with mental illness and suicidal gestures since she was a young teenager. The significance of our findings is discussed in the context of the substantial lack of current research on SDOH in transgender individuals to underscore the need for clinical awareness and promote future research.

## Introduction

The World Health Organization (WHO) defines social determinants of health (SDOH) as “the circumstances in which people are born, grow up, live, work and age, and the systems put in place to deal with illness.” These systems are further shaped by “economics, social policies, and politics” on the national and local levels leading to societal hierarchy and health inequities [1]. The social determinants are intimately connected to non-medical factors that influence the health outcomes of many individuals. It is possible that when they negatively influence health, mental illness can develop. For example, limited access to education limits work opportunities leading to low income and poverty which is associated with mental illness.

Transgender (trans) individuals are those who experience gender incongruence between their personal gender identity and the gender they have been assigned at birth. Before publishing the Diagnostic and Statistical Manual of Mental Disorders, Fifth Edition, transgenderism was a psychiatric diagnosis of “Gender Identity Disorder,” which has since been replaced with “Gender Dysphoria,” a step toward reducing the stigma surrounding transgenderism [2,3]. Transgenderism is a gender identity that can sometimes be mistaken to mean intersex or sexual orientation. A person who is intersex possesses both male and female biologic characteristics (chromosomes, gonads, genital traits, etc.), whereas sexual orientation is determined by sexual preference. Transgender people can be sexually attracted to the same sex or the opposite sex, identifying as homosexual or heterosexual, respectively [4].

Multiple factors make it difficult to estimate the size of the transgender population in the United States or around the world. Transgender people are a diverse group of individuals but only a small number of them are receiving gender-affirming treatments at health clinics where they are accessible to researchers. While those who purchase their hormones on the streets or the internet transition only socially without accessing any gender-affirming treatments and live in parts of the world where stigma or a potential threat to their life prevents them from making their transgender status known are difficult to quantify and remain inaccessible to researchers. Therefore, researchers mainly study the populations who visit gender-affirming clinics which underestimates the proportion of the transgender population at large [4]. Moreover, health surveillance systems in the United States do not routinely include gender identity as a standard measurement to help quantify transgender populations [5]. This makes it more difficult to not only quantify transgender people accurately but also places limitations on comparison studies between trans and cisgender groups to evaluate SDOH. In 2016, it was estimated that 0.5-1.3% of birth-assigned males and 0.4-1.2% of birth-assigned females worldwide were transgender, averaging about 25 million transgender individuals globally [4]. These numbers are too high to be taken lightly.

This case report illustrates how SDOH can affect mental health in transgender patients. We highlight the prevalence and impact of low education rates, poverty, unemployment, financial instability, sex work, homelessness, and access to healthcare within the transgender population.

## Case presentation

We present the case of Ms. XX, a 22-year-old, single, unemployed, undomiciled, African American transgender female who was admitted for a suicide attempt during a methamphetamine overdose. She was brought to the emergency room (ER) by emergency medical services, activated by herself because she had plans to jump into the oncoming traffic.

On evaluation in the inpatient psychiatric unit, Ms. XX reported that she had planned to overdose on crystal meth and jump into the oncoming traffic but “chickened out.” She reported that during her most recent methamphetamine intoxication, she felt “scarred, paranoid, like people were following me all the time.” The patient endorsed auditory hallucinations of voices stating mean things directed toward her but she was unable to recall details of what voices had said about her. The patient stated that the voices were more than one person, they were unfamiliar, and only present when she was intoxicated from crystal meth. She denied auditory hallucinations of command type instructing her to hurt herself or others. She reported that auditory hallucinations during methamphetamine intoxication only started within the last couple of months. The patient was aware that the voices and paranoid delusions were because of crystal meth intoxication and did not occur at any other time; however, these psychotic episodes caused her a lot of distress. The patient endorsed a depressed mood, anhedonia, poor sleep and appetite, and feelings of worthlessness for more than two weeks. The patient reported that she intended to overdose on crystal meth with plans to move into oncoming traffic because of family abandonment, homelessness, and lack of financial support.

Ms. XX stated that around five to six years of age was the first time when she felt “different.” She reported that she used to tell her cousins “I have female blood going through my body” and that was how she described her attraction to boys. She first thought of wanting to die at 14 years of age because of the “confusion and stress” caused by her gender dysphoria. She also reported experiencing periods of elevated, euphoric mood, with an increase in goal-directed activities and decreased need for sleep at an unknown age. At 14 years of age, she first started feeling helpless, hopeless, and worthless. She reported becoming intoxicated with alcohol at a family party and once sober she felt “depressed and guilty” and thought of wanting to kill herself. The patient reported having no specific plan. She reported cutting her wrists during that same year with the intent to die and was admitted to an inpatient psychiatric hospital for the first time. The patient was unable to recall the length of stay, specific diagnosis, or name of the hospital. She reported being discharged with Prozac of unknown strength but Prozac was discontinued at the request of her mother for unknown reasons. She was also unable to recall if outpatient follow-up care was provided. The patient’s family could not be contacted for collateral information. Ms. XX reported that her last hospitalization was at age 21 years. She reported being “triggered” after a verbal altercation with a fast food employee at a drive-through after she was referred to as “sir.” The patient reported immediate thoughts of wanting to jump off a bridge but got scared and called 911. She reported never physically going to a bridge. She was sent to the hospital but later transferred to a psychiatric facility. The patient was admitted for about two to three days and was discharged on Lexapro and Abilify of unknown strength and quantity. She was unable to recall any past diagnosis given or if any follow-up appointments were provided at that time.

Ms. XX reported being kicked out of her family’s home at 20 years of age because of her decision to transition to the female gender. She stated she had the option to live with her grandmother but she decided to travel instead. She moved from a suburban neighborhood to a major metropolitan city with no financial or family support. She explained that she coped with her stress by traveling and partying. She reported feeling the most depressed during that time. She reported having fatigue and decreased energy despite sleeping more than 12 hours a day. The patient also reported having anxiety “because I always thought everyone was judging me, but I realized later that no one really cared.” The patient had poor social support networks.

Ms. XX reported completing a high school education. She reported working in a clothing store in her hometown for one to two years before leaving her family home. She also worked at a fast food chain for less than one year but was unable to maintain employment due to unclear reasons, possibly due to substance abuse and mental illness. Currently, the patient reported supporting herself through sex work and living at a shelter.

The patient reported increased self-medication by smoking methamphetamines and cannabis. She started using methamphetamines three to four months ago daily and was unable to quantify the amount but said she used it anytime she could get it. She last smoked meth one day before her current admission. She reported taking the substance in large amounts, spending a lot of time getting, using, or recovering from it. She reported cravings and urges to use the substance. She reported continuing to use despite the impairment of relationships and despite the dangers it had caused her. She reported needing more of the substance to get the same effect as before. Ms. XX also reported alcohol use starting at 14-15 years of age. She reported drinking “a couple” of Hennessey drinks per week, and her last drink was one week before admission. She denied binge drinking, blackouts from alcohol intoxication, or seizures relating to alcohol withdrawal. Moreover, Ms. XX reported increased cannabis use. She also reported tobacco use starting at 14-15 years of age. She reported smoking approximately “a few” cigarettes weekly and stated she “hardly ever smokes cigarettes.” Her last cigarette was one day ago before admission.

Medical management during psychiatric inpatient hospitalization involved starting the patient on Lexapro 10 mg PO daily and Abilify 10 mg PO daily. Estradiol 2 mg PO BID and spironolactone 50 mg PO daily were also added and managed per the medical team. After clinical improvement of her symptoms, the patient was discharged with Abilify Maintena 400 mg IM with referrals to a therapist, psychiatrist, and general medicine physician.

## Discussion

Research has shown that the transgender population is associated with higher rates of poverty, unemployment, homelessness, substance use, psychological distress, suicide, and overall mental illness [6]. It is possible that social determinants are more important in influencing health than traditional known factors, and when these factors negatively influence health, mental illness can develop.

Ms. XX finished high school but did not attempt college. She reported being bullied, isolated, and alienated by her peers. The only time she felt accepted and respected by her peers and family was during her brief hypermasculine phase at 14-15 years of age when she worked out a lot and grew out facial hair in an attempt to fit in. Education is a key SDOH that provides tools for people to fight poverty by increasing the chances of employment and stable income. It allows for food and housing security and reduces socioeconomic and political inequities [7]. However, 32% of transgender men and 22% of transgender women reported having less than a high school education level [8]. Such high numbers could be attributed to feeling unsafe in their environment, provided that 77% of transgender responders (U.S. Transgender Survey in 2015) reported being verbally harassed, prohibited from expressing their gender identity through clothing, and physically or sexually assaulted on more than one occasions from kindergarten to grade 12. About 17% of transgender people reported dropping out of school and 6% were expelled [6]. In the 2017 cycle of the Youth Risk Behavioral Survey, 1.8% of grade 9-12 students across ten states and nine large urban school districts in the United States identified as transgender. These transgender students reported a higher prevalence of violence victimization (including forced sexual intercourse and physical dating violence), substance use, and suicide risk than their equivalent cisgender male and female students [9]. More than 70% of transgender individuals reported being discriminated against in schools [10]. Transgender students reported feeling unsafe in schools compared to their cisgender peers due to increased bullying. As a result, transgender students are more likely to skip school and exhibit chronic absences [11]. Numerous studies have shown that victimization, social isolation, and bullying are associated with increased substance use, depression, anxiety, and suicide risks in youth minority groups [12,13]. It is important to create a safe and supportive environment for transgender kids in schools because education has significant implications for transgender individual health.

Moreover, Ms. XX’s low education level reduced her employment opportunities and exposed her to low-paying jobs and financial instability. Reportedly, her most recent employment was at a fast food chain for less than one year which she was unable to maintain due to unclear reasons. Transgender individuals face high levels of poverty, unemployment, and economic instability. The rate of poverty among transgender individuals is two times higher than the national average. The rate of unemployment is three times higher [6]. Transgender individuals have a multitude of difficulties retaining employment. Interpersonal conflicts, discrimination which was reported by nearly 70% of transgender employees [10], and harassment eventually lead to unemployment. The study showed that workplace discrimination is 2.5 times more likely to occur in gender-diverse employees (transgender, non-binary, genderqueer) than in cisgender men [14]. Despite the growing literature on workplace discrimination and harassment, comparative research on transgender individuals is lacking at large.

The harsh reality of financial instability and unemployment is that transgender individuals are turning to sex work for income. Unfortunately, Ms. XX became a victim of this statistic when she turned to sex work after she was unable to maintain stable employment. About 12% of transgender individuals have reported having sex for income in the United States, while another study from Puerto Rico reported more than 42% of transgender responders have engaged in survival work sex [6,10]. Transfemale sex workers (TFSW) are at four times the risk of HIV infection than cisfemale sex workers, with an overall prevalence of 27.3% of HIV infections in TFSW and 4.5% in cisfemale workers [15]. In addition to the increased risk of sexually transmitted infections, sex work may also predispose individuals to increased rates of violence against them and mental illness.

Ms. XX was kicked out of her family’s home when she was 20 years old due to her new transgender status. She was forced to abandon her hometown and moved to a larger metropolitan city where she faced homelessness and found refuge in a shelter. Housing is an essential physiological need and a key SDOH for all people. The U.S. Transgender survey in 2015 reported that nearly 30% of transgender individuals face homelessness in their lifetime, 23% were evicted or denied housing based on gender discrimination in the past year, and 26% avoided staying in shelters out of fear. As many as 70% of transgender people living in shelters reported various forms of mistreatment because of their gender identity [6].

Homelessness and mental illness have a complex temporal relationship that has not been studied well in the context of transgender individuals. It is difficult to establish causality because in some cases mental illness precedes homelessness while precipitating or aggravating it in others. The prevalence of homeless transgender youth living on the streets is significantly higher than that of homeless cisgender youth [16]. Homelessness is associated with an increased risk of HIV in transgender people in the United States [17], which complicates disease management and is a strong predictor of poor health outcomes [18,19].

Furthermore, Ms. XX reported not having an outpatient medical provider, psychiatrist, or therapist. She also could not recall any past diagnosis given and if any follow-up appointments were given to her after previous psychiatric hospitalizations. The patient denied past medical and surgical histories. She denied past family psychiatric history, family substance use history, and family history of suicide attempt or completion. She was in increased need of psychiatric and therapy services but was unable to access them. Moreover, Ms. XX reported being 20 years old when she first received gender-affirming care in the form of estradiol and spironolactone; however, she was not consistent with it due to her limited access to medical care. The last time she received hormonal medications was during her last psychiatric hospitalization. Access to quality health care is another significant SDOH in the context of the transgender population. This minority population experiences higher rates of substance use, suicide rates, and mental illness. The use of marijuana, illicit substances, and non-medical prescription drugs is nearly three times that of the U.S. population. The rate of suicide attempts is nine times higher than in the general population, which means 42.6% of transgender individuals attempted suicide in their lifetime [6]. About 59.2% of transgender individuals reported using mental health services due to depression, eating disorders, and suicidal behavior in 2015 [10]. In Canada where there is universal healthcare, transgender individuals used health services (especially for mental health needs) at higher rates than the general population. Transgender individuals have higher rates of chronic illnesses such as HIV, diabetes, asthma, and chronic obstructive pulmonary disease than cisgender individuals [20]. Despite an increased need for quality medical care in the transgender population, 85.7% of transgender individuals reported being unable to cover healthcare costs, with only 62.2% having publicly funded healthcare coverage, and 88.5% considering it difficult to access both medical and transgender healthcare services [10].

## Conclusions

Many factors contribute to the health outcomes of transgender individuals. Through an in-depth examination of the case report presented, several significant SDOH influencing mental health in urban transgender patients were elucidated. First, low education rates, unemployment, and housing instability were critical determinants of overall physical and mental health outcomes. Second, poor social networks in the form of family abandonment, isolation, and discrimination were fundamental determinants with significant negative impacts on mental health. Lastly, maladaptive coping strategies exacerbated ongoing and predisposed to future mental illness in the absence of appropriate health care and mental health services. It is vital to address the SDOH to reduce health disparities and promote health equity among transgender persons in urban areas. Understanding the prevalence and impact of unemployment, education, sex work, homelessness, substance use, suicidality, violence, and discrimination in transgender individuals is critical. It is important for clinicians to understand the various challenges transgender patients face. Awareness of the complex domains of SDOH and unique medical considerations for transgender individuals is essential when formulating a comprehensive treatment plan.
